# Adjunctive Use of Statins for COVID-19

**DOI:** 10.3390/jcm10071407

**Published:** 2021-04-01

**Authors:** Mike Sathekge, Geert Byttebier, Bart De Spiegeleer, Bo Saxberg, Veronica Ueckermann, Luc Belmans, Myriam Alexander, David Fedson

**Affiliations:** 1 Department of Nuclear Medicine, University of Pretoria & Steve Biko Academic Hospital, 0002 Pretoria, South Africa; 2 Nuclear Medicine Research Infrastructure (NuMeRI), University of Pretoria, 0002 Pretoria, South Africa; 3 Bioconstat BV, 9000 Ghent, Belgium; geert.byttebier@skynet.be; 4 Drug Quality and Registration Group, Faculty of Pharmaceutical Sciences, Ghent University, 9000 Ghent, Belgium; Bart.DeSpiegeleer@ugent.be; 5 DDO Strategic Services, Tucson, AZ 85737-8488, USA; bo@saxberg.com; 6 Department of Internal Medicine, University of Pretoria & Steve Biko Academic Hospital, 0002 Pretoria, South Africa; veronica.ueckermann@up.ac.za; 7 Medaman bv, 2440 Geel, Belgium; luc.belmans@medaman.be; 8 Open Health, Marlow, Buckinghamshire SL7 2FF, UK; MyriamAlexander@openhealthgroup.com; 9 57, Chemin du Lavoir, 01630 Sergy Haut, France; davidsfedson@gmail.com

The interaction between obesity, cardiometabolic disorders and COVID-19 represents a syndemic that requires both social intervention and a multipharmacological approach. The risks associated with diabetes, obesity and hypertension for severe COVID-19 may be confounded by the type of medication for these cardiometabolic factors. Furthermore, endothelial dysfunction is a common feature of the key comorbidities that increase risk for severe COVID-19 such as hypertension, obesity, diabetes mellitus, coronary artery disease or heart failure [1,2,3]. Among the drugs used to manage hypertension and diabetes mellitus are angiotensin-converting enzyme (ACE) inhibitors, angiotensin receptor blockers (ARBs) and statins, all of which are known to improve endothelial dysfunction [1,2].

Several therapeutic agents have already been assessed for treating COVID-19, but few of them have been shown to be efficacious [4]. The lack of treatment options is largely due to limited information on other potential treatments and the lack of multicentre clinical trials evaluating their safety and efficacy [5].

Statins are commonly used to treat high-risk patients and are known to reduce cardiovascular morbidity and mortality [1,3,5]. They are highly effective because of their dual role in the clearance of cholesterol from intracellular and extracellular compartments. Statins inhibit 3-hydroxy-3-methyl glutaryl (HMG)-CoA reductase, a rate-limiting enzyme that catalyses the conversion of HMG-CoA into L-mevalonate 1, and thereby inhibit cholesterol biosynthesis [5,6]. Importantly, statins also inhibit the synthesis of isoprenoids, which are vital to cell function. Inhibition of prenylation accounts for much of their anti-inflammatory activity [5,7,8].

Recent evidence also suggests that statins inhibit virus infection by (a) decreasing L-mevalonate downstream mediators, (b) inhibiting protein prenylation, and (c) upregulating angiotensin-converting enzyme 2 [5,8]. These mechanisms underscore the importance of lipid metabolism in the pathogenesis of virus infections. Cholesterol and lipid metabolism are essential for several steps in the life cycle of many viruses, including virus fusion to cell membranes and cell entry, virus replication and particle assembly, and virus release from host cells. Host lipid metabolic pathways are known to promote the replication and infectivity of several viruses [9].

A compelling example was provided by the unconventional and poorly documented use of a statin and angiotensin receptor blocker combination to treat patients with Ebola [10]. Treatment was followed by a significant decrease in mortality, presumably by restoring endothelial cell function and supporting tissue repair [10,11].

There is rapidly growing evidence supporting the potential benefit of treating COVID-19 patients with statins [2,9,12,13,14,15,16,17]. Importantly, the use of statins correlates significantly with lower mortality in patients with COVID-19, consistent with the findings in patients with pneumonia (Figure 1). Furthermore, the National Institutes of Health COVID-19 Treatment Guidelines recommend that patients who are already taking statins for the treatment or prevention of cardiovascular disease should continue statin treatment if they develop COVID-19 [18].

However, there are no data or prospective trials to support the idea of starting de novo statin therapy in patients with mild and moderate COVID-19 with the aim of preventing its progression to more severe disease. There is also limited information on the potential drug–drug interactions, particularly with drugs that are metabolized by the same cytochromes as statins.

In view of their record of efficacy and affordability, the adjunctive use of statins is an attractive option for treating COVID-19 patients. We urge investigators to undertake additional observational studies and clinical trials of statins, ACE inhibitors, and ARBs in these patients.

## Figures and Tables

**Figure 1:**
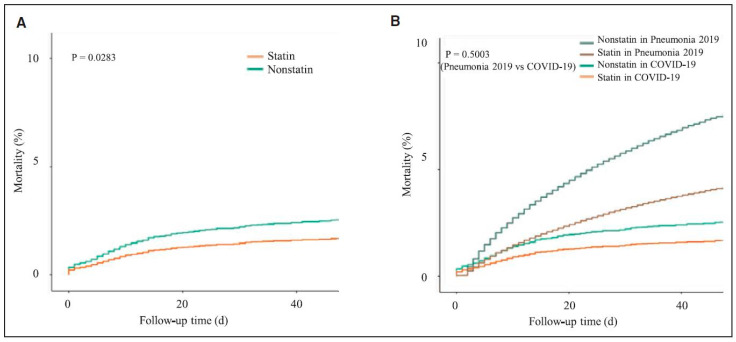
Survival within 60 d in severe acute respiratory syndrome coronavirus 2-infected patients and a retrospective cohort of hospitalized pneumonia patients between January and June 2019 (overall cohort). (**A**) Comparison of statin-treated vs. statin-nontreated coronavirus disease 2019 (COVID-19) patients (adjusted). (**B**) Comparison of statin-treated vs. statin-nontreated COVID-19 and hospitalized pneumonia patients between January and June 2019 (retrospective cohort, adjusted) [17].

## References

[1] Nägele M.P., Haubner B., Tanner F.C., Ruschitzka F., Flammer A.J. (2020). Endothelial dysfunction in COVID-19: Current findings and therapeutic implications. Atherosclerosis.

[2] De Spiegeleer A., Bronselaer A., Teo J.T., Byttebier G., De Tré G., Belmans L., Dobson R., Wynendaele E., Van De Wiele C., Vandaele F. (2020). The effects of ARBs, ACEis, and statins on clinical outcomes of COVID-19 infection among nursing home residents. J. Am. Med. Dir. Assoc..

[3] Fedson D.S., Opal S.M., Rordam O.M. (2020). Hiding in plain sight: An approach to treating patients with severe COVID-19 infection. mBio.

[4] Khan A.R., Misdary C., Yegya-Raman N., Kim S., Narayanan N., Siddiqui S., Salgame P., Radbel J., Groote F., Michel C. (2021). Montelukast in hospitalized patients diagnosed with COVID-19. J. Asthma.

[5] Parihar S.P., Guler R., Brombacher F. (2019). Statins: A viable candidate for host-directed therapy against infectious diseases. Nat. Rev. Immunol..

[6] Istvan E.S., Deisenhofer J. (2001). Structural mechanism for statin inhibition of HMG-CoA reductase. Science.

[7] Jain M.K., Ridker P.M. (2005). Anti-inflammatory effects of statins: Clinical evidence and basic mechanisms. Nat. Rev. Drug Discov..

[8] Greenwood J., Steinman L., Zamvil S.S. (2006). Statin therapy and autoimmune disease: From protein prenylation to immunomodulation. Nat. Rev. Immunol..

[9] Vuorio A., Kovanen P.T. (2021). Statins as adjuvant therapy for COVID-19 to calm the stormy immunothrombosis and beyond. Front. Pharmacol..

[10] Fedson D.S., Jacobson J.R., Rordam O.M., Opal S.M. (2015). Treating the host response to Ebola virus disease with generic statins and angiotensin receptor blockers. mBio.

[11] Fedson D.S. (2016). Treating the host response to emerging virus diseases: Lessons learned from sepsis, pneumonia, influenza and Ebola. Ann. Transl. Med..

[12] Bifulco M., Gazzerro P. (2020). Statin therapy in COVID-19 infection: Much more than a single pathway. Eur. Heart J. Cardiovasc. Pharmacother..

[13] Castiglione V., Chiriacò M., Emdin M., Taddei S., Vergaro G. (2020). Statin therapy in COVID-19 infection. Eur. Heart J. Cardiovasc. Pharmacother..

[14] Dashti-Khavidaki S., Khalili H. (2020). Considerations for statin therapy in patients with COVID-19. Pharmacotherapy.

[15] Aparisi Á., Amat-Santos I.J., López Otero D., Marcos-Mangas M., González-Juanatey J.R., San Román J.A. (2021). Impact of statins in patients with COVID-19. Rev. Esp. Cardiol. (Engl. Ed.).

[16] Sorice M., Misasi R., Riitano G., Manganelli V., Martellucci S., Longo A., Garofalo T., Mattei V. (2021). Targeting Lipid Rafts as a Strategy Against Coronavirus. Front. Cell Dev. Biol..

[17] Lee H.Y., Ahn J., Park J., Kyung Kang C., Won S.H., Wook Kim D., Park J.H., Chung K.H., Joh J.S., Bang J.H. (2021). Beneficial Effect of Statins in COVID-19-Related Outcomes-Brief Report: A National Population-Based Cohort Study. Arterioscler. Thromb. Vasc. Biol..

[18] National Institutes of Health COVID-19 Treatment Guidelines 2020. https://www.covid19treatmentguidelines.nih.gov/concomitant-medications/.

